# Good exams made easy: The item management system for multiple examination formats

**DOI:** 10.1186/1472-6920-12-63

**Published:** 2012-08-02

**Authors:** Achim Hochlehnert, Konstantin Brass, Andreas Möltner, Jobst-Hendrik Schultz, John Norcini, Ara Tekian, Jana Jünger

**Affiliations:** 1 Department of Psychosomatic and General Internal Medicine, University of Heidelberg, Medical Clinic, Heidelberg, Germany; 2 Centre of Excellence for Medical Assessment, University of Heidelberg, Medical School, Heidelberg, Germany; 3 Foundation for Advancement of International Medical Education and Research (FAIMER), Philadelphia, PA, 19104, USA; 4 College of Medicine at Chicago, University of Illinois, Chicago, IL, 60612, USA

**Keywords:** Assessment alliance, Quality control

## Abstract

**Background:**

The development, implementation and evaluation of assessments require considerable resources and often cannot be carried out by a single faculty/institution. Therefore some medical faculties have founded cooperation projects which mainly focus on the exchange of multiple choice questions (MCQs).

**Methods:**

Since these cooperation projects do not entirely support all relevant processes in terms of preparation, implementation and evaluation of assessment, in 2006 the Medical Assessment Alliance (MAA) was founded for mutual support. In addition to MCQs the MAA started to develop innovative assessment formats and facilitate content through a coordinated exchange of experiences. To support cooperation within this network, the web-based Item Management System (IMS) was developed which supports all processes of the assessment workflow as an all-in-one working platform.

**Results:**

At present, the Alliance has 28 partner faculties in Europe. More than 2.800 users in 750 working groups are collaborating. Currently 90.000 questions have been stored in the IMS. Since 2007, nearly 4.600 examinations have been successfully conducted.

**Conclusion:**

This article describes in detail the unique features of the IMS and contrasts it with the item management systems of other associations.

## Background

Although medical faculties have considerable freedom in designing their assessments [1], the development, implementation, and evaluation of such assessments represent a huge challenge. The design of “good” exams not only includes the creation of tasks [2] but also conducting examinations, reviewing examination papers for content and accuracy, defining the grading system, statistically analyzing the exams, and reporting results. Many faculties use multiple-choice questions because moving forward to more innovative question types is supposed to be a time-consuming and expensive procedure. In the same way, the implementation of review processes in many faculties is proving very difficult due to limited resources [3]. Automated exam correction, grading, statistical analysis, the use of document reading systems, evaluation programs, or the implementation of computer-based assessments have resulted in resource savings. However, for question design, quality assurance of the tasks and exam blueprints there has been only limited progress.

In English-speaking countries there are consortia which have developed item pools to share assessment content (e.g., IDEAL, an international coalition of faculties [4] with an item database which currently contains approximately 32.000 items (personal communication with Prof. R. Hays, May 2012) or UMAP, a coalition of medical schools in Great Britain [5]). While they include, for example, the development of common standards, they do not cover all areas of assessment design and implementation in comparable depth (e.g., detailed exam blueprints and implementation of a pre-review [6]).

The Medical Assessment Alliance (MAA) represents, in contrast to the IDEAL and UMAP consortia, a significant development in using a shared web-based platform, called the Item Management System (IMS). This supports staff in the preparation, implementation, evaluation, and reporting of assessments according to existing guidelines.

This article describes the MAA/IMS and critically evaluates experience with it, future challenges, and possible solutions.

## Medical assessment alliance

**Figure 1:**
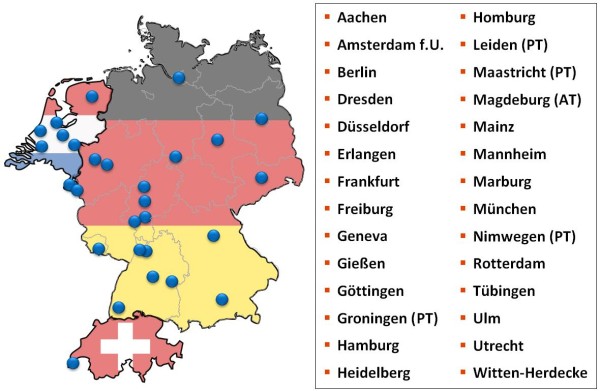
**Medical faculties involved in the medical assessment alliance.** Four faculties in the Netherlands have chosen to use the IMS for progress testing (PT), the German faculty of Magdeburg uses the IMS for admission tests (AT).

In 2006 the medical faculty of Heidelberg, the Charité Berlin and the LMU Munich set up the MAA [7] to facilitate cooperation and joint problem solving in assessment which occur against the backdrop of limited resources. Under the leadership of the Centre of Excellence for Medical Assessments, objectives were set out in a cooperation agreement. The item bank contains not only MCQs, but also key features, OSCE, and structured oral exams [8]. Since its inception, 28 medical faculties have joined the Alliance (see Fig. 1):

There are annual two-day consensus meetings with the leaders of staff responsible for assessments from all partner faculties. These, for example, jointly develop innovative assessment formats, exchange OSCE checklists, and construct guidelines for the review process. Joint decisions are made on awarding external contracts, adjusting processes, and improving the user-friendliness of the system. In 2010 and 2011 the following topics were addressed in Alliance meetings: Review activity, development of a composite internal progress test and selection tests, exchange of OSCE checklists, and the development of encounter cards.

Since regular formative feedback promotes competency-based teaching [9], the Alliance is also involved in collaborative research projects which support the faculties (e.g., formative assessments and progress test [10]).

To strengthen inter-faculty collaboration, the web-based IMS was implemented. It is an all-in-one platform which supports the work of staff responsible for the preparation, administration, evaluation and reporting of assessments [11]. The cost of operating and developing this platform is shared by the entire Alliance as a non-profit organization.

## Description of the IMS assessment platform

### Current figures on the IMS assessment platform

#### Use of the assessment platform

Currently 2.800 users are registered with IMS who are working together in 750 groups. In these groups, people are no longer just limited to their own faculty but have found inter-faculty colleagues with whom they design items and examinations and cooperate on other aspects of assessment processes.

To date 4.600 examinations composed of a total of 123.000 questions have been successfully administered to more than 800.000 medical students using IMS. 584.000 statistical test parameters (e.g. item difficulty, selectivity, etc.) have been calculated and these help in the compilation of future exams. These have been uploaded to the IMS database.

#### Development of the exam database

**Figure 2:**
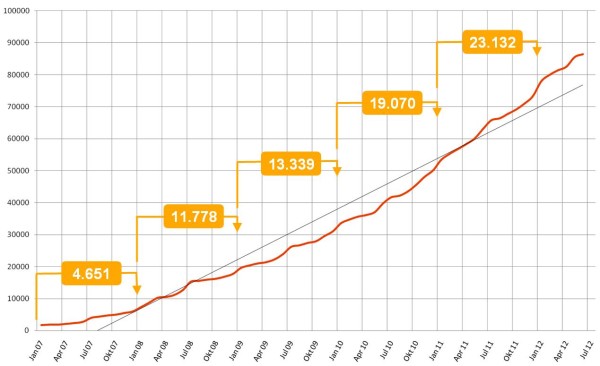
**Development of the number of items in the database.** The development of the item numbers in the database shows a continuous increase. The numbers in brackets indicate the increase per year.

**Figure 3:**
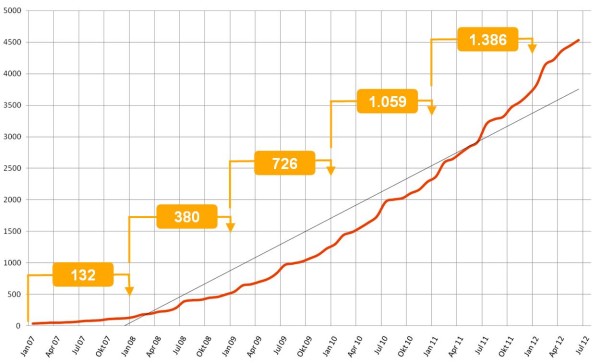
**Development of the number of examinations in the database.** The development of the number of examinations conducted with the IMS. The numbers in brackets indicate the increase per year

The database stores more than 90.000 questions and a number of these can be made accessible to other faculties by the item author. Currently 19% of all questions are available to all users of the Alliance for their exams. Figure 2 shows the development of the number of test items in the database over a period of five years. Figure 3 shows the number of examinations conducted with the IMS.

### Description of the individual IMS features

Each IMS user receives their own protected user account and can access the IMS based on clearly defined roles and responsibilities with a personalized view. To support collaborative work, colleagues can set up working groups and design exams as a team or conduct quality control processes.

In IMS, the complete assessment sequence is laid out as described by the Assessment Committee of the German Medical Association (Gesellschaft für Medizinische Ausbildung GMA) and the guidelines of the Centre of Excellence for Medical Assessments [6]. In the following, the eight key components of this process are explained:

#### Task design

Assessment content (items) can be created and saved in IMS in various formats, including long menu, free text, MCQs, key feature cases, OSCE checklists, and structured oral examinations [12]. Entering the test items is supported by format-specific input forms. Graphics and figures can be easily integrated into the questions; the integration of sound and video material will soon be available.

#### Task classification in IMS

In addition to classification by subject and sub-subject, every faculty can create their own classification system in the IMS to classify tasks according to their specific learning targets. This allows, for example in the case of a Swiss Faculty, for classification into a Bachelor’s and Master’s system which is differentiated into several course units and modules. The implementation of a classification system linked to the German National Competency-based Learning Targets Catalogue in Medicine (NKLM) [13] is in progress.

#### Task evaluation prior to assessment (pre-review)

According to existing guidelines, all examination questions should be evaluated in a pre-review prior to their use in a live assessment [6]. This can be done in the IMS using a standardised review checklist which was jointly designed by the Alliance. The review can be carried out by colleagues either individually or as groups [14], which means that more than two reviewers come together in a meeting and review items directly in the IMS.

**Figure 4:**
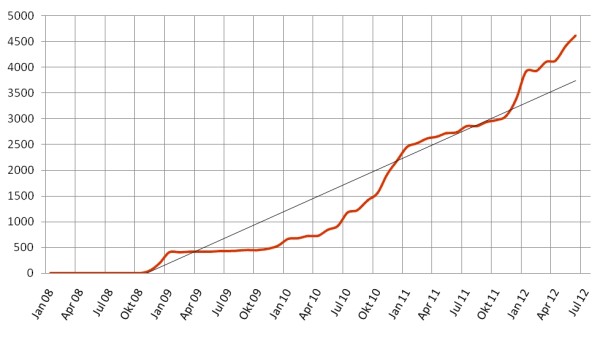
**Development of review activity.** Following a new release of the IMS in March 2010 which involved significant improvements of system functionalities a clear increase of the review activity can be seen.

Figure 4 shows the evolution of review activity after switching from a peer review process to a more user-friendly assessment process and presenting the review results in a visually appealing way in IMS.

#### Collating exam tasks

Examinations can easily be assembled by dragging and dropping questions into exams. Apart from tasks created personally by the authors, they also have access to a public pool available to all Alliance members.

To further promote efficiency in the organisation of assessments, it is possible to choose various blueprint sub-functions. For example, 1) a table of contents blueprint can be used for targeted exam compilation (e.g., defining the proportion of cardiology tasks in an exam on internal medicine), 2) an evaluation blueprint can be used to define the distribution of points, and 3) a date blueprint can be used to monitor all examinations of a faculty over a fixed period of time (e.g. for compiling an exam in a timely manner before the exam date).

#### Compiling an assessment

IMS allows the user to create printable exam sheets (in PDF format). The items can be permuted using flexible algorithms to create several versions of an examination which could be sorted by subject, item-type, etc.. In addition, standard interfaces for transferring data to computer-based assessment programs or document readers are integrated.

#### Conducting an assessment

An examination can be conducted on paper, as traditionally the case, computer-supported using machine-readable answer sheets, computer-based in a computer lab, as an oral exam, or as an OSCE. By using IMS interfaces, each partner faculty can use its own established systems or standard Alliance tools, such as the document reading system Klaus® [15] or the computer-based exam system Campus® [16], which are available free of charge to Alliance members.

#### Evaluation

Following the examination, the raw data can be evaluated using IMS. The system has interfaces for importing this data from a document reading system or a computer-based examination system. The system calculates scores for subtests and total test scores based on parameters which can be individually set, such as the maximum number of points of a question or the grading algorithm (e.g. application of automatic adjustment rules, etc.). IMS also calculates detailed test statistics, including question and exam indicators such as difficulty, accuracy or the index of discrimination [17].

#### Post-review

Potentially wrong tasks can be identified through conspicuous statistical test parameters. In such a case, the points awarded in such tasks can be corrected as part of a post-review or tasks can be removed from the overall exam score. Such information is stored in the IMS in order to document the deficiencies and to facilitate revision by the author. Furthermore, comments by teaching staff or test participants can be recorded.

**Figure 5:**
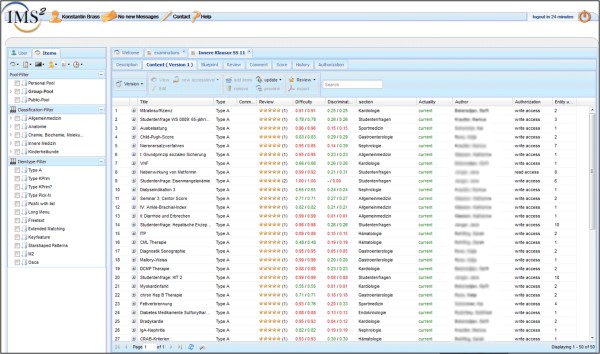
**Item management system graphical user interface (excerpt).** The navigation toolbar is on the left, on the right the item details can be viewed, such as the item’s summary, the item type, information on difficulty and discriminatory power and the review results, etc.. For clarity and for privacy reasons details regarding the author, sharing etc. are omitted or blanked.

Figure 5 gives an impression of the graphical user interface of the IMS where all this information is clearly represented.

Apart from direct support for the aspects listed above, IMS offers further infrastructure assistance to study deaneries such as monitoring upcoming exams and keeping a record of actors used as standardized patients.

#### Actor database additional module

The IMS also provides support for the development and implementation of standardised objective clinical or oral-practical examinations (e.g. OSCE). The organisation and management of actor-patients is often an additional challenge due to the large number of people involved. The module integrated into IMS for this purpose can be used to manage events, assign roles, and reimburse the actor-patients. In addition to its use in OSCE examinations, this module can also be used to organise the use of actor patients in the teaching of the regular curriculum. This not only frees up considerable resource in the planning of teaching activities but also improve quality assurance.

## Discussion

Exchanging assessment experiences and exam tasks created at other faculties form an important basis for improving medical education. The use of a web-based platform to support assessment significantly eases such cooperation. Both the MAA and the IMS assessment platform are in line with the *Consensus Statement and Recommendations from the 2010 Ottawa conference* which states that “the medical education community still needs to develop a deeper understanding of how technology can underpin and extend assessment practices” [18].

Within 5 years it has been possible to establish a successful and stable inter-faculty cooperation among 28 faculties. Despite differences in the human and technical resources of the partner faculties, MAA has a high acceptance which, amongst other things, is clearly demonstrated by the speed with which it has grown to include 28 participating faculties and the intensive use of the system. The accession of the medical faculty of Geneva as a French-speaking university and of the faculties in the Netherlands as Dutch-speaking universities is a further signal that the IMS not only offers extra value due to its extensive collection of questions (which for the most part available in German only) but is a complete system with a high degree of functionality and the workflow assistance. This differentiates the IMS from other known assessment associations such as the IDEAL Consortium, which focuses mainly on building a database of exam questions [4] and UMAP, which involves a commitment to the quality assurance of tasks. A faculty conducts quality assurance reviews as a contribution and in return receives access to a certain number of reviewed tasks. However, a department can only view the descriptors of each question prior to using it, not the question itself [5]. Other international networks are either not focused on assessment (FAIMER [19,20] or do not support the entire assessment process using IT [21].

One of the advantages of the IMS is that authors can decide whether they want to share items with others. Some people choose not to publish their items because they only want them to be used locally. The full benefit of the system will be realized as authors begin to see the quality and efficiency inherent in cross faculty collaboration.

The analysis of user experience shows that the further development of the IMS promotes quality development processes at participating faculties. For example, the review activity of IMS participants increased significantly during the past year following changes to the process which included e.g. more flexible ways to perform a review of an item or a better presentation of the review results. To further expand review activity, cooperation in specialised assessment associations (e.g. paediatrics, occupational health) is consistently promoted and supported by the MAA (e.g., advice, presentations at professional conferences, and help with networking).

A technical challenge is the development of powerful search algorithms. At present, more than 17.000 questions are managed in the shared pool and the number is rising steadily. Using additional filters which take statistical test parameters, review results, usage frequencies of the tasks, and MeSH terminology into account [22], there are plans to increase the efficiency of the search functions in future to further improve access.

Currently, in spite of the variety of question formats (long menu, free text, MCQs, key feature cases, OSCE, etc.) available in the IMS, the main type used are MCQs (96%). This mirrors results which showed that MCQs are still the predominant format at German medical faculties [2]. In this regard, the IMS offers the possibility of supporting further implementation of urgently needed competency-based assessment formats and content due to its support features for practical and oral assessments.

The development of competency-based assessment formats and the creation of the relevant content are increasingly seen as a major challenge internationally. Qualification frameworks like the competencies developed by the Accreditation Council for Graduate Medical Education (ACGME) [23] or the National Competency-based Learning Targets Catalogue in Medicine (NKLM) for Germany [13] which are in line with the CanMEDS roles [24] will contribute to giving further impetus to competency-based assessment. As these competency-based Catalogues are applied nationwide, the individual learning targets can be deposited in the IMS and linked directly with the exam papers. Additionally, the Alliance regularly submits joint applications for joint research projects, for example focussing on the question of how to integrate other test formats which are now on the horizon. To catalyse these changes, the German Federal Ministry of Education and Research has funded a five-year project starting 2012 to develop competency-based examination formats and generate content.

In the past, assessments at individual faculties have focused on summative examinations. It is crucial for steering the students’ learning process to receive feedback on their current knowledge through formative tests. For example, the progress test is a format, growing in popularity, demonstrates growth in knowledge over time [25]. As the creation and evaluation of progress tests is very resource intensive for a single faculty, the MAA has already done several steps to realize this project as a joint endeavour together with the faculty of Maastricht (Netherlands).

The concept of having many partners who contribute to improving quality by providing their special knowledge is one of the main reasons for the success of MAA. Through the consensus meetings and the direct exchange between doctors, psychologists, and IT developers, the system enhances the quality development processes at the faculties (see Table 1). Additionally the participation in the MAA provides economic benefits for each participating faculty because this way of cooperating directly results in savings for every partner.

Over a three year period, nearly 450.000€ were needed to develop the first version of the IMS. Following this, the development and maintenance of the IMS is financed by the fees of the participating faculties. The costs for one faculty in the pilot phase is 10 € per student. Thereafter, the cost per year is 30 € per student to a maximum of 25.000 € per year per faculty. This includes organizational and technical support as well as further development of the IMS. Participating faculties benefit economically because they do not have to develop a similar system but only pay for the running costs of the IMS.

## Conclusion

Through the cooperation of various university partners, the task of creating fair, high quality, competency-based assessments can be shared. Overall, the MAA has fulfilled the expectations in terms of the creation and design of exams. The increase in the number of users and the continuous increase in the number of items suggest that the IMS has been accepted and is used extensively. The IMS has proven itself as a practical and efficient solution for the entire assessment workflow, so that new challenges can be explored and addressed.

## Competing interests

The authors report no conflicts of interest. The authors alone are responsible for the content and writing of this article. Authors guarantee that the manuscript has not been published elsewhere and it has not been submitted simultaneously for publication elsewhere. Data concerning the number of items, examinations, review etc. has been updated on the occasion of resubmission and represents the status by June 30^th^ 2012.

## Authors’ contributions

AH specialised in health technology assessment and evaluates the impact of Informatics on Medical Education. He drafted the manuscript and participated in the coordination of the project. KB implemented the first version of the IMS and coordinates the development of the IMS-project. AM provided the acquisition of the data. JJ and JHS are responsible for the medical education program at the Medical hospital and conceived the project. JN and AT participated in the finalization of the manuscript and provided valuable feedback to the IMS and the manuscript. All authors read and approved the final manuscript.

## Pre-publication history

The pre-publication history for this paper can be accessed here:

http://www.biomedcentral.com/1472-6920/12/63/prepub
